# *Lacticaseibacillus paracasei*: Occurrence in the Human Gut Microbiota and *K*-Mer-Based Assessment of Intraspecies Diversity

**DOI:** 10.3390/life11111246

**Published:** 2021-11-17

**Authors:** Maria Frolova, Sergey Yudin, Valentin Makarov, Olga Glazunova, Olga Alikina, Natalia Markelova, Nikolay Kolzhetsov, Timur Dzhelyadin, Viktoria Shcherbakova, Vladimir Trubitsyn, Valery Panyukov, Alexandr Zaitsev, Sergey Kiselev, Konstantin Shavkunov, Olga Ozoline

**Affiliations:** 1 Laboratory of Functional Genomics and Cellular Stress, Federal Research Center “Pushchino Scientific Center for Biological Research of the Russian Academy of Sciences”, Institute of Cell Biophysics of the Russian Academy of Sciences, 142290 Pushchino, Russia; mosmasha@mail.ru (M.F.); glazuova.olga.a@gmail.com (O.G.); alikina.olga@mail.ru (O.A.); markelova.n.y@gmail.com (N.M.); kolya.kolzhecov@mail.ru (N.K.); dzhelyadin@pbcras.ru (T.D.); panyukov@itaec.ru (V.P.); anthyllium@gmail.com (S.K.); 2 Centre for Strategic Planning of Federal Medical-Biological Agency of Russia, 119121 Moscow, Russia; info@cspmz.ru (S.Y.); makarovvalentine@gmail.com (V.M.); 3 Laboratory of Anaerobic Microorganisms, Federal Research Center “Pushchino Scientific Center for Biological Research of the Russian Academy of Sciences”, Institute of Biochemistry and Physiology of Microorganisms of the Russian Academy of Sciences, 142290 Pushchino, Russia; shcherb@ibpm.pushchino (V.S.); lichoradkin43@gmail.com (V.T.); 4 Institute of Mathematical Problems of Biology RAS—The Branch of Keldysh Institute of Applied Mathematics of the Russian Academy of Sciences, 142290 Pushchino, Russia; sasha-z@psn.ru

**Keywords:** *Lacticaseibacillus paracasei*, *k*-mer-based phylogeny, human gut metagenomes, Crohn’s disease, autism, obesity, antibiotic therapy, probiotics

## Abstract

Alignment-free approaches employing short *k*-mers as barcodes for individual genomes have created a new strategy for taxonomic analysis and paved a way for high-resolution phylogeny. Here, we introduce this strategy for the *Lacticaseibacillus paracasei* species as a taxon requiring barcoding support for precise systematics. Using this approach for phylotyping of *L. paracasei* VKM B-1144 at the genus level, we identified four *L. paracasei* phylogroups and found that *L. casei* 12A belongs to one of them, rather than to the *L. casei* clade. Therefore, we propose to change the specification of this strain. At the genus level we found only one relative of *L. paracasei* VKM B-1144 among 221 genomes, complete or available in contigs, and showed that the coding potential of the genome of this “rare” strain allows its consideration as a potential probiotic component. Four sets of published metagenomes were used to assess the dependence of *L. paracasei* presence in the human gut microbiome on chronic diseases, dietary changes and antibiotic treatment. Only antibiotics significantly affected their presence, and strain-specific barcoding allowed the identification of the main scenarios of the adaptive response. Thus, suggesting bacteria of this species for compensatory therapy, we also propose strain-specific barcoding for selecting optimal strains for target microbiomes.

## 1. Introduction

The *Lactobacillus casei* group of bacteria, consisting of the closely related *L. casei*, *L. paracasei*, *L. rhamnosus* and *L. zeae* are among the most studied lactobacilli due to their commercial, industrial, and applied health potential [1]. They are often used to ferment dairy products conferring improved flavor and texture. It has also been found that bacteria of this group produce a variety of bioactive metabolites that can benefit the host when consumed [2]. Lactic acid bacteria *L. paracasei* are widely distributed in nature [3] and are found on plant materials, from where they enter the human microbiome, usually being localized in the oral cavity and gastrointestinal tract.

However, the taxonomic history of lactobacilli has been confusing and complex. Overall, by March 2021, the genus *Lactobacillus* included 261 species that are extremely diverse at the phenotypic, ecological and genotypic levels and perhaps more than other taxons require new strategies for phylotyping. In a study by Zheng et al. [4], the phylogeny of *Lactobacillaceae* and *Leuconostocaceae* was investigated based on complete genome sequences. The authors assessed a large set of classification parameters including such criteria as pairwise mean amino acid identity, the presence of clade-specific genes and the phylogeny of the conserved genome core. According to this classification, 13 species were combined into a new genus *Lacticaseibacillus*, which includes *Lacticaseibacillus paracasei*. In this paper, we will follow this classification introducing *Lacticaseibacillus paracasei* subsp. *paracasei* VKM B-1144 as a newly characterized isolate and evaluate its relatives.

Our previous study [5] showed that *k*-mer-based phylogenetic analysis, operating with thousands of marker sequences without any limitation from the level of genome functional annotation, could be efficiently used for intraspecific systematics. Such a taxonomy is of obvious importance for species containing pathogenic strains/phylogroups and, vice versa, for potential probiotics. Up to now, thorough intraspecific phylotyping has been done mostly for *Escherichia coli* [6,7,8,9,10,11,12,13,14,15] and several other species from the human intestinal microbiome [16,17]. Here we employed this novel technique for bacteria with probiotic potential and showed their distribution in human gut microbiota depending on the health status, antibiotic/probiotic treatment or dietary shift.

## 2. Materials and Methods

### 2.1. Bacterial Growth

Bacteria were grown on an anaerobic medium of the following composition (g/L): casein peptone, 10.0; meat extract, 10.0; yeast extract, 5.0; glucose, 20.0; Tween 80, 1.0; K_2_HPO_4_, 2.0; sodium acetate, 5.0; ammonium citrate, 2.0; MgSO_4_ × 7 H_2_O, 0.2; MnSO_4_ × H_2_O, 0.05; the pH of the medium was 6.2–6.5. For solidification of the medium, Bacto agar 1.5% (w/v) was added. The medium was prepared using the Hungate anaerobic technique [18]. Cultivation was carried out in 15 ml Hungate tubes or on Petri dishes placed in anaerobic jars (Oxoid Limited, Basingstoke, UK). Bacterial cultures were incubated at 37 °C for 24–48 h until the appearance of individual colonies or the onset of the early stationary growth phase.

### 2.2. DNA Preparation

Genomic DNA was isolated from bacteria cultured in liquid medium at the stationary growth phase. Since *L. paracasei* are known to possess a thickened cell wall, we used a specific protocol for its effective disruption [19]. Following centrifugation, the cell pellet was washed with 30 mM NaCl and 2 mM EDTA, pH 8.0. The cells were subsequently incubated in buffer (pH 8.0) containing 2 mM EDTA, 20 mM Tris-HCl, 20 mg/mL lysozyme, and 1% Triton X-100 for 2 h at 37 °C. For more efficient lysis, 10 mg/mL of RNase A and 20 mg/mL of proteinase K were added and incubated for one more hour at 55 °C. Finally, a Genomic DNA Purification Kit (Promega, Madison, WI, USA) was used for DNA clean-up according to the manufacturer’s protocol. The concentration of DNA was measured using a Nanodrop ND-1000 spectrophotometer (Thermo Scientific, Wilmington, DE, USA).

### 2.3. Genome Sequencing

Genome sequencing of the studied isolate was carried out using the Ion Torrent PGM platform (Thermo Fisher Scientific, Waltham, MA, USA). A library for sequencing was prepared using the NEBNext Fast DNA Fragmentation and Library Prep Set for Ion Torrent (NEB, Ipswich, MA, USA), according to the manufacturer’s instructions. The concentration of the resulting library was evaluated on a Qubit 3 fluorometer (Thermo Fisher Scientific, Waltham, MA, USA) using Qubit dsDNA HS Assay Kit reagents (Thermo Fisher Scientific, Waltham, MA, USA). The library was amplified using an Ion One Touch 2 sample preparation system and Ion PGM Hi-Q View OT2 Kit (Thermo Fisher Scientific, Waltham, MA, USA). Sequencing was performed with an Ion 316 Chip Kit v2 BC (Thermo Fisher Scientific, Waltham, MA, USA). Fastq files were filtered on the Galaxy server [20] using the “Filter by Quality” option (parameters: Q20 and coverage 90%) and ignoring all reads with degenerated nucleotides. The genome was resequenced on a MinION device (Oxford Nanopore Technologies, Oxford, UK) using a SQK-LSK109 kit and MinKNOW software v. 21.02.1, following the Genomic DNA by Ligation protocol.

### 2.4. Genome Assembly and Annotation

Combined sets of reads obtained from the Ion Torrent PGM and Oxford Nanopore MinION were assembled *de novo* using facilities of the All Bacterial Bioinformatics Resource Center PATRIC [21]. The resulting genome was annotated using the RAST server [22]. For the analysis of glycoside-active enzymes, we used the data from the Carbohydrate-Active Enzymes database [23]. CAZY proteins were annotated using the dbCAN resource [24]. The analysis of transport systems was carried out using the database TransportDB 2.0 [25].

### 2.5. Barcoding

In order to barcode genomes by marker *k*-mers (*k* = 18), we used a local copy of the NCBI GenBank database as of February 19, 2020, containing 39,822 nucleotide sequences of fully assembled bacterial genomes and plasmids, including 75 genomes of *Lacticaseibacillus* genus together with strain VKM B-1144. When searching for genomes closest to VKM B-1144 we also analyzed *L. paracasei* sequences assembled in contigs or scaffolds. For phylogenetic analysis, a set of unique 18-mers was obtained for each genome of the *L. paracasei* species, residing in its sequence and/or in the sequences of other strains of the genus of *Lacticaseibacillus* (or species *L. paracasei*), but absent in the genomes of all other bacteria and plasmids from the database. For taxonomic analysis of the human intestinal microflora, all 18-mers present in the genome of *Homo sapiens* were also removed from the obtained sets. All the procedures were carried out in the virtual machine with a 64-bit version of UniSeq software on a high-performance server (configuration: 2 Xeon Gold 5218, 64 Gb RAM) [5,26].

### 2.6. Phylogenetic Analysis

After sets of marker 18-mers present only in *L. paracasei* strains (species level) or in genomes of *L. paracasei* and bacteria of closely related species (*L. rhamnosus*, *L. casei* and *L. manihotivorans*) were obtained, their pairwise comparison was done and the Sørensen similarity distances were calculated [27]. Phylogenetic trees were constructed using the neighbor-joining [28] and minimum evolution [29] methods in MEGA X [30].

### 2.7. Phylogroup-Specific Taxonomic Analysis of Human Metagenomes

Metagenomes were obtained from NCBI Short Read Archive (SRA) and included four sets of metadata [31,32,33,34], listed in the Supplementary Table S1. Each dataset contained whole genome shotgun sequence reads of metagenomes obtained from fecal samples. Two sets allowed the possibility of comparing the presence of *L. paracasei* strains in the microbiomes of healthy individuals and patients with chronic disease (children with autism spectrum disorders (PRJEB23052) [31] and patients with Crohn’s disease (PRJNA290380) [32]). Two other sets provided the possibility for pairwise comparisons of metagenomes taken from a particular individual before and after treatment. In the first one, fecal samples were collected from overweight/obese persons before and after adherence to Mediterranean dietary restrictions (PRJEB33500) [33], and persons before and after antibiotic treatment either subjected to subsequent probiotic therapy or not (PRJEB28097) [34]. Fastq files obtained from the European Nucleotide Archive (ENA) and NCBI SRA were trimmed for the adaptor sequence and quality controlled with Trim Galore v.0.6.6 [35]. The read length cutoff was set for 20 b and low-quality reads with a Phred less than 20 were excluded.

The metagenomes were transcoded the same way as described in [5], i.e., (**A**), (**C**), (**G**), and (**T**) were substituted with the numerals (**0**), (**1**), (**2**) and (**3**), respectively, while line breaks were replaced with the numeral (**4**). As a result, metagenomes were digitized in one numeric string, which allowed use of the UniTestExpress software [5] to operate them in the same way as the genomes. Comparing genomes and metagenomes, there is the possibility to combine *k*-mers unique for different genomes into a cumulative set and remove common *k*-mers from the two metadata to be compared. In this study we used species-specific sets of 18-mers, obtained in each genome of *L. paracasei* but absent in bacterial genomes of other species or genera. We also used strain-specific 18-mers absent in all the genomes of the local copy of the NCBI GenBank database except for the sequence of the target genome. The option “Clear” was used to remove cross-linking *k*-mers from the target sets. As in our previous study [5], the number of sequence reads containing marker 18-mers, rather than the number of unique 18-mers *per se*, was used for the statistical analysis. Since reads are rarely identical, this helps to avoid multiple counting of unique *k*-mers that make up long tracks, while retaining the possibility of quantitative assessment. Since in some cases the collected data did not show a normal distribution, the non-parametric Mann–Whitney U test was used in all cases to estimate the statistical significance of the differences between two groups.

## 3. Results

### 3.1. Characteristics of the L. paracasei VKM B-1144 Strain

*Lacticaseibacillus paracasei* subsp. *paracasei* strain VKM B-1144 (other identifiers: ATCC 25303, CCM 1752, NCIMB 700152, NIRD H831 and C6) [36,37,38], originally isolated from the human oral cavity [39,40], was obtained from the All-Russian Collection of Microorganisms (IBPM RAS, Pushchino, Russia). The cells of the strain are Gram-positive rods 2–4 µm in length and 0.8–1.0 µm in width. They are seen as single cells during exponential growth in a liquid medium, but form long chains in the stationary phase. On solid medium, the bacteria form whitish colonies with a diameter of 2–3 mm.

### 3.2. Genome Sequencing and Assembly

The genome of *L. paracasei* VKM B-1144 was primarily sequenced using the Ion Torrent PGM platform. A total of 2,621,451 sequence reads with an average length of 245 bases were obtained, which gave 2,041,237 sequences after filtration by quality on the Galaxy server [20]. The 16S rRNA gene sequence of the bacterium was determined with primers 27f (5′-AGAGTTTGATCCTGGCTCAG-3′) and 1492r (5′-TACCTTGTTACGACTT-3′) and phylogenetic analysis allowed identification of *L. paracasei* strain EG9, having the genome of 2,927,257 b.p. in length, as the closest relative. In view of this, semiconductor sequencing provided an approximately 174-fold genome coverage. In order to improve the draft genome assembly, the strain under study was resequenced using an Oxford Nanopore MinION device, which yielded 90,066 reads, with 15,381 reads filtered out for poor quality by the software. Thus, the resulting nanopore set included 73,806 reads, which varied in length in the range from 112 to 78,113 b.p., achieving a ~105-fold genome coverage. Four scaffolds were obtained, with the longest sized 2,921,318 b.p. Comparison of the shotgun whole genome with the genomes of *L. paracasei* strains JCM 8130 and EG9, showed the average nucleotide identity (ANI) to be 98.5% and 98.6%, respectively, which confirmed the results of 16S rRNA phylotyping. The genome under study was compared with the two abovementioned genomes to scaffold smaller contigs manually based on the positions of gene sequences, thus providing the second scaffold with a length of 24,996 b.p. 

### 3.3. Genome Annotation and Analysis

**Figure 1:**
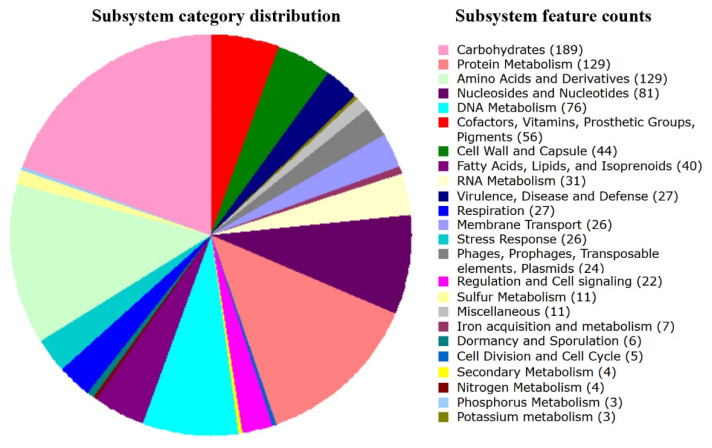
Color diagram obtained as a result of genome-wide RAST annotation, showing the distribution profile of the identified coding sequences according to their belonging to different functional categories. Only gene sets with identified subsystems are indicated.

Genome annotation was performed using the RAST server [22] with the identification of 3043 sequences of protein coding genes. Figure 1 demonstrates their distribution over the main functional categories.

As expected, a significant part of the genes found in the genome are associated with carbohydrate metabolism (189 genes). Their analysis in the dbCAN2 database [24], providing automated annotation of carbohydrate-active enzymes, showed that the genome of the VKM B-1144 strain encodes a wide range of glycoside-active enzymes, such as glycoside transferases, glycoside hydrolases, as well as polysaccharide lyases and carboxyesterases (GH1, GH2, GH3, GH13, GH20, GH25, GH29, GH31, GH32, GH35, GH36, GH65, GH73, GH88, GH136, GH170). For example, there are genes encoding proteins homologous to glucan 1,6-α-glucosidase, α-phosphotrehalase, oligo-1,6-glucosidase; neopullulanase and 1,4-α-glucan branching enzyme, structurally belonging to the GH13 family.

All of these proteins act on the α-1,4- and α-1,6-glycosidic bonds present in starch, glycogen and related oligosaccharides such as maltose. In addition, glycogen synthase (GT5) and glycogen phosphorylase (GT35) were found among the transport proteins. This indicates that the VKM B-1144 strain can accumulate cellulose on the cell wall surface in the form of an extracellular matrix, which facilitates cell adhesion and biofilm formation protecting cells from extracellular stress factors [41,42,43]. Moreover, lactobacilli often synthesize glycogen as a storage substance and use it to interact with a wide variety of environments [44].

Four large categories in Figure 1, quite expectedly, include genes encoding proteins involved in nucleic acid and protein biosynthesis, while the next largest set of protein-coding genes (56) corresponds to the biogenesis of cofactors, vitamins, prosthetic groups and pigments. In this connection, it is important that the value of probiotic bacteria in the human intestine is largely determined by their ability to synthesize vitamins and cofactors *de novo* and supply them to the host. The human gut microbiota is capable of synthesizing vitamin K and most of the group B vitamins, such as pyridoxine, folate, riboflavin, cobalamin, and thiamine. Among them, thiamine (vitamin B1) as thiamine pyrophosphate (TPP) plays a critical role in host energy metabolism, since it acts as a cofactor in major metabolic pathways, such as the pentose phosphate pathway, glycolysis and the Krebs cycle. The pentose phosphate pathway is required for the synthesis of steroids, nucleic acids, fatty acids, and the biosynthesis of aromatic amino acids. These products are used as precursors for the biogenesis of various neurotransmitters and other bioactive compounds vital for brain function [45]. Most of them can be produced by the VKM B-1144 strain. Although, the genome of these bacteria does not have a complete set of genes for thiamine biosynthesis, there are genes for the thiamine salvage pathway.

Extracellular proteins with the LPXTG motif deserve particular attention. They can be attached to the cell wall by sortases and play an important role in the mechanisms of probiotic action [46], facilitating cell adhesion and habitat colonization. According to the data of Ghosh et al. [47], the number of proteins with the LPXTG motif differs from 0 to 12 among the studied *L. paracasei* strains. In the genome of VKM B-1144 we found at least three genes for such proteins: outer membrane protein Lmo 0159, peptidoglycan protein Lmo2821 and von Willebrand factor type A (Lmo 2576), but their number can increase up to nine if protein-coding potency is confirmed for six other genomic loci. The presence of three genes encoding sortase A indicates the ability of VKM B-1144 bacteria to successfully overcome the mucous barrier and colonize the intestinal tract, thus supporting their value as potential probiotics.

### 3.4. Intrageneric and Intraspecific Phylotyping of L. paracasei Genomes

Using the standard nomenclature [48], adopted after the updated classification of bacteria from the *Lacticaseibacillus* genus, 76 genomes were taken from the NCBI database. This set included 43 *L. paracasei* chromosomes and 33 chromosomes of closely related species, of which 26 belonged to *L. rhamnosus* strains, 6 to *L. casei*, and 1 to the *L. manihotivorans* LM010 strain. It turned out that there are three assembly versions (AP011548.1, CP031290.1 and FM179322.1) for the *L. rhamnosus* GG (or ATCC 53103) genome in the database. Therefore, to avoid duplication, only one genome was taken for phylogenetic analysis (accession number CP031290.1). The remaining 74 genomes of the *Lacticaseibacillus* strains, along with the VKM B-1144 assembled chromosome, are listed in Table 1 and are characterized in more detail in Supplementary Table S2.

**Figure 2:**
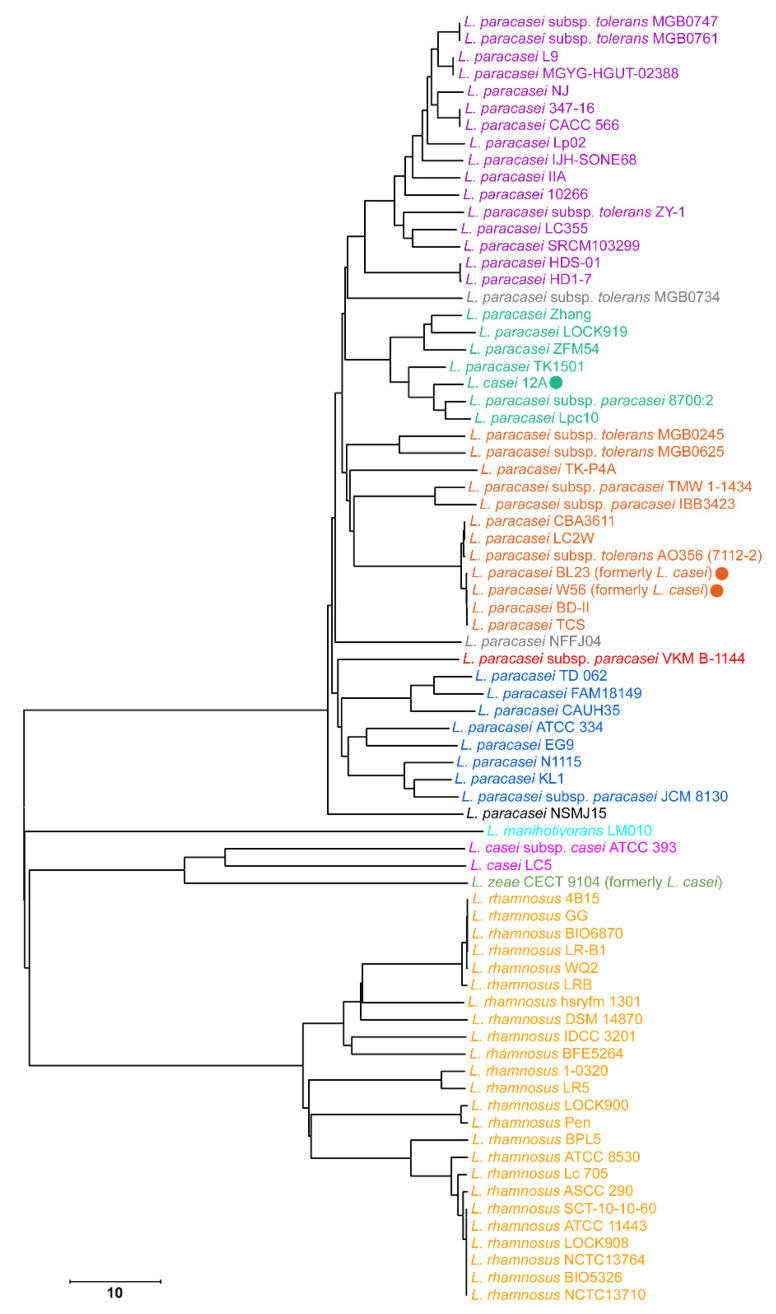
Phylogenetic tree constructed by the neighbor-joining method in MEGA X software [30]. The tree was inferred from the pairwise distance matrix for 75 sets of 18-mers unique to the genus *Lacticaseibacillus*. Strains BL23 and W56, previously ascribed to the *L. casei* species, as well as *L. casei* 12A, are marked by circles. The clades/branches of species *L. rhamnosus*, *L. casei*, *L. zeae* and *L. manihotivorans*, as well as four main *L. paracasei* phylogroups, are marked with different colors. Two branches for MGB0734 and NFFJ04 in the common clade of phylogroups I–III are shown in gray; the isolate VKM B-1144 is highlighted in red, and the outbreak from the whole *L. paracasei* clade (NSMJ15) is in black. The scale bar shows the Sørensen distance as a percentage.

For phylotyping at the intrageneric level, the sets of unique genus-specific 18-mers were obtained for all 75 genomes using the UniSeq software [5]. For this, all 18-mers that belong to chromosomes other than those of the genus *Lacticaseibacillus* according to the modern classification, as well as to all plasmids, were removed from the database. These 75 individual sets (barcodes) belonging to the genomes of the same genus overlap to various extents in pairwise comparison, which allows us to use them for phylogenetic analysis. Using the numbers of common 18-mers for each pair of 75 genomes as a measure of their similarity, we calculated a pairwise distance matrix [27], as described in Materials and Methods. Phylogenetic trees were constructed using the neighbor-joining and minimum evolution methods. Since they were independent of the method, both for representatives of the genus *Lacticaseibacillus* and for the species *L. paracasei*, only the tree obtained by neighbor-joining [28] in MEGA X [30] is shown in Figure 2.

For *L. paracasei* bacteria this tree has four large clades containing 7–15 species. This intraspecies heterogeneity is in line with the data of Smokvina et al. [49], who characterized the diversity of *L. paracasei* based on multilocus alignment of 183 protein-coding sequences of the core genome compiled from 34 laboratory strains. The set of genomes analyzed in that paper included only three genomes available in the NCBI Database (Zhang, BL23 and ATCC 334). The same as in our case (Figure 2), they belonged to different clades. Thus, although the search for differences in metabolic traits and evolutionary pathways for different clades requires further analysis, it became clear that *k*-mer-based intraspecies phylogeny works reproducibly even for a rather small set of compared genomes.

In addition to four large clades, four single branches are clearly separated within the *L. paracasei* cluster. They are: *L. paracasei* subsp. *tolerans* MGB0734, *L. paracasei* NFFJ04, *L. paracasei* subsp. *paracasei* VKM B-1144 and *L. paracasei* NSMJ15, of which the latter strain outgrouped in relation to all the rest of the strains of *L. paracasei*. We also found that the chromosomes of strains BL23 and W56 (clade III) and 12A (clade II) (indicated by circles in Figure 2), that were taken from the NCBI GenBank as *L. casei*, cluster together with *L. paracasei*.

It turned out that the chromosomes of these three strains had a lower GC content (46.3%–46.4%), which is typical for *L. paracasei*, and not for *L. casei* (47.9%–48%). Thus, it is reasonable that in November 2020, the specification of BL23 and W56 in the NCBI GenBank was changed to *L. paracasei*. However, strain 12A is still referred to as *L. casei*. According to our data, its classification should also be reconsidered.

Since the strain CECT 9104 formerly annotated as *L. casei* is currently annotated as *L. zeae*, only two strains with complete genomes (ATCC 393 and LC5) remained in the *L. casei* clade. The genomes of *L. rhamnosus* strains, on the contrary, form a single large cluster in full accordance with the annotation, and the genome of *L. manihotivorans* LM010, as expected, occupies a branch independent of other species (Figure 2).

**Figure 3:**
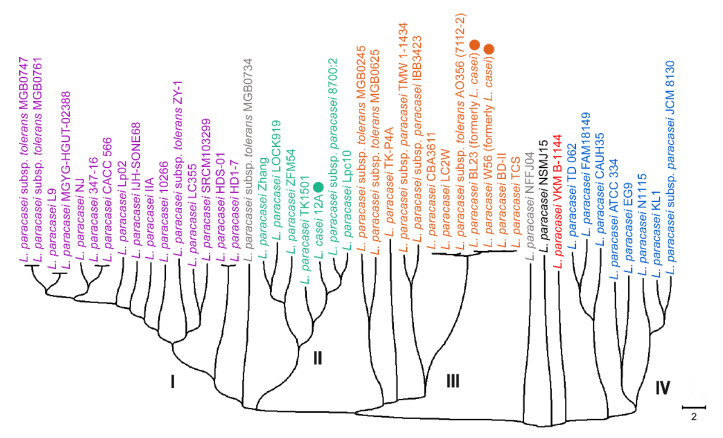
Phylogenetic tree constructed by the neighbor-joining method in MEGA X software [30]. The tree was inferred from the pairwise distance matrix for 47 sets of 18-mers unique to the species *Lacticaseibacillus paracasei*. The color code is the same as in Figure 2. The scale bar shows the Sørensen distance as a percentage.

The two next steps were implemented to estimate the stability of the tree obtained for *L. paracasei* genomes. At the first step, we checked the diversity of *L. paracasei* at the species level. For this purpose, 18-mers present only in the genomes of *L. paracasei* strains were obtained (Figure 3). The topology of the tree remained virtually unchanged, except that the *L. paracasei* NSMJ15 strain ceased to be an outgroup in relation to the rest of the clades.

The topology and composition of all four large clades, as well as the presence of all singletons, including *L. paracasei* VKM B-1144, appeared to be completely reproduced. Thus, at the second step we searched for microorganisms related to our strain among all the genomes in the NCBI GenBank, which are annotated as *L. paracasei*, although deposited as sets of contigs or scaffolds rather than complete genomes. As of 11 October 2021, there were 175 of them (Supplementary Table S3), and *k*-mer-based phylogeny allows us to use their species-specific 18-mers together with 47 complete genomes similarly to Figure 3.

The resulting tree constructed for 222 strains is shown in Supplementary Figure S1. The groups I, II and IV are well separated and increased. The group III, which previously included 12 strains, subdivided into three subgroups, of which the first one captured the MGB0734 strain that previously formed an individual branch between groups I and II (marked in gray in Figure 2 and Figure 3). A new phylogroup was identified between groups I and II, which includes the NFFJ04 genome (indicated by a gray circle in Supplementary Figure S1) and seven more genomes assembled in contigs. The NSMJ15 strain shows some homology only with strain SRCM103410, while *L. paracasei* VKM B-1144 demonstrated a higher homology with only *L. paracasei* DSM 20207 and these two pairs form clades with the earliest divergence from the main phylogroups. Thus, the next part of this study was aimed at understanding: how evenly different phylogroups of *L. paracasei* are distributed in the human microbiota, and how much their presence depends on the physiological state of an individual.

### 3.5. Phylogroup-Dependent Profiling of L. paracasei Presence in Human Intestinal Microbiomes

Four sets of freely available metadata with a total of 117 human gut microflora shotgun metagenomes were used for this analysis (listed in Supplementary Table S1). Two sets allowed a comparison of the presence of *L. paracasei* strains in the microbiomes of healthy individuals and patients with a chronic disease. Of these, autism spectrum disorder (20 samples) was selected as a target pathology due to the increased attention to the gut microbiota in its treatment [31,50,51], and Crohn’s disease (14 samples) since it is well known to cause intestinal dysbiosis [5,32,52]. In both cases the percentage of sequence reads containing species-specific 18-mers of *L. paracasei* genomes was averaged over control samples (10 and four metagenomes, respectively) and samples obtained from patients with autism spectrum disorder (ASD) or Crohn’s disease (10 metagenomes in each case).

In the case of samples obtained from overweight/obese persons, 50 metagenomes were available for comparison, 25 of which were obtained from individuals before adherence to Mediterranean dietary restrictions and 25 after the nutritional plan [33]. Samples were taken from 13 men and 12 women aged from 21 to 65 years. The most promising was the last group with 33 fecal samples taken from 11 healthy antibiotic-naive individuals before and after receiving ciprofloxacin and metronidazole for 7 days, followed by 8 weeks of recovery with probiotics treatment or without it (Supplementary Table S1). Among 11 bacterial species, the probiotic cocktail contained *L. paracasei* bacteria [34].

Metagenomes were prepared as described in Materials and Methods and species-specific sets of marker 18-mers were obtained to assess the level of *L. paracasei* strains in fecal samples. Their numbers, indicated in columns four and nine of Table 2, ranged from 1,000,877 (TD 062 strain genome) to 1,250,212 (IBB3423). Thus, the percentage of reads found in metagenomes was normalized per one million marker 18-mers.

**Figure 4:**
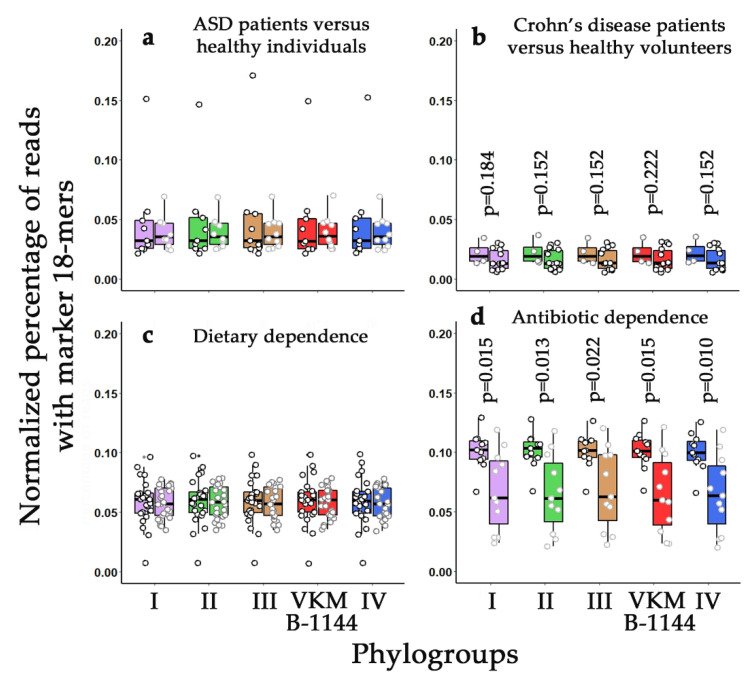
Dependence of the presence of *L. paracasei* bacteria on the health status (**a**,**b**) or acquired treatment (**c**,**d**). Circles mark the presence of species-specific 18-mers of 45 genomes in different metagenomes. For phylogroups I–IV, they are averaged over 8–16 genomes (Table 2). Box plots reflect their variability. Data from healthy or untreated individuals (control samples) are plotted on the left in each of the grouped pairs. Boxplots were produced in R using the ggplot2 package.

In natural metagenomes this percentage reflects the presence of close relatives for each genome. The values obtained for 44 genomes were averaged over four phylogroups indicated in Figure 3 and Table 2 (Figure 4). For strain VKM B-1144 they are shown independently (red plots in Figure 4).

It became clear, that *L. paracasei* make up about 0.05% of the human intestinal microflora. Their presence is fairly consistent across the given datasets. Variability in the range from 0.02% to 0.1% is most likely due to the difference in methods used by the authors for DNA extraction, because peptidoglycan walls of Gram-positive bacteria require more severe treatment for cell disruption. For all that, we removed one metagenome from the selected metadata because of an abnormally high abundance of *L. paracasei* in the control sample of the first set (about 4%). This means that such variations do happen.

**Figure 5:**
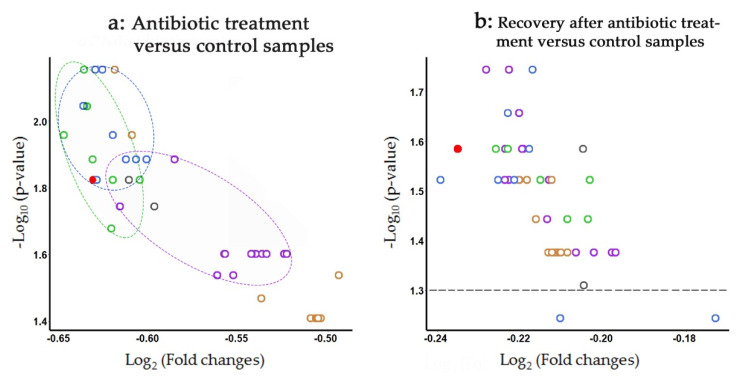
Volcano plots, reflecting fold change ratios in the number of reads with species-specific marker 18-mers from 47 genomes of L. paracasei in 33 metagenomes in response to a 7-day antibiotic treatment of 11 healthy volunteers (**a**) and the effectiveness of microbiome recovery over 8 weeks (**b**). The second set of metagenomes was composed of 5 samples taken from persons who received a probiotic cocktail during the first 28 days and from 6 individuals who recovered spontaneously (Supplementary Table S1). The color code is the same as in Figure 2, Figure 3 and Figure 4. Symbols corresponding to NFFJ04 and NSMJ15 are gray; colored ovals highlight close localization of groups I, II and IV symbols on panel (**a**). The dashed line on panel (**b**) indicates the cut-off level for statistical significance corresponding to a 0.05 probability of getting a false result.

We did not observe any changes in the presence of *L. paracasei* in the metagenomes of children with ASD syndrome (Figure 4a) and in response to dietary shift (Figure 4c). The level of *L. paracasei* in the intestinal microflora of patients with Crohn’s disease tended to decrease (Figure 4b), but this effect was not statistically significant. However, in response to antibiotic administration, the intestinal abundance of *L. paracasei* was significantly reduced and turned out to be more variable than in the control samples (Figure 4d). Although all phylogroups exhibited highly similar changes, the response of bacteria from the second and fourth groups was statistically more significant. Therefore, at the next step, we compared fold ratios in the number of reads with the marker 18-mers for all 47 genomes in the metagenomes of 11 people in response to antibiotic treatment (volcano plot in Figure 5a).

It became clear that bacteria belonging to phylogroups II and IV (green and blue ovals in Figure 5a) are on average more sensitive to antibiotics than bacteria of the two other groups. In line with the phylogenetic trees (Figure 2 and Figure 3), the strain VKM B-1144 also belongs to the same category. The bacteria of phylogroup III demonstrated the highest diversity in terms of antibiotic sensitivity, which might be symptomatic, because this group subdivided into several clades in Supplementary Figure S1. Probably, the most surprising observation in this part of the study is shown in Figure 5b: even after a long recovery period, the abundance of *L. paracasei* bacteria remains significantly suppressed in the microbiota of people subjected to antibiotic treatment.

### 3.6. Strain-Specific Characterization of Antibiotic/Probiotic-Mediated Changes in Microbiomes

Hoping to increase the sensitivity of *k*-mer-based taxonomy, in the next part of the study we used strain-specific 18-mers, which do not overlap with the barcodes of other known genomes of *L. paracasei* strains. Their number is much lower than that of species-specific 18-mers (Table 2), and in two cases (strains L9 and MGYG-HGUT-02388) removal of 18-mers present in at least one other genome yielded empty sets. This outcome is explained by the presence of closely related species in our reference set (Table 2) and can be avoided by selecting one of them for withdrawal, which is difficult to substantiate. Hence, in this study we removed 16 genomes with the smallest barcode sets from the analysis. The rest of the sets (bolded in Table 2) ranged from 11,840 to 86,180 18-mers in size, with an average number of 36,794. Thus, the percentage of reads with barcodes of 31 genomes found in metagenomes was normalized per 37,000.

**Figure 6:**
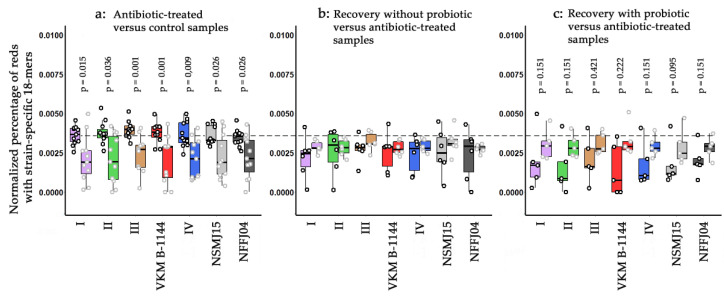
Dependence of the presence of L. paracasei bacteria in the human intestine on the antibiotic treatment (**a**), spontaneous recovery (**b**) and probiotic therapy (**c**). Circles show the presence of strain-specific 18-mers of 31 genomes in different metagenomes. For phylogroups I-IV, they are averaged over 5–9 genomes (Table 2). Box plots reflect their variability. Data are shown by paired box plots, of which the left in panel (**a**) corresponds to samples taken from 11 healthy antibiotic-naïve individuals, while panels (**b**,**c**) reflect abundance of *L. paracasei* in metagenomes sampled from the same persons, who received probiotic therapy (5 samples) or not (6 samples). The dashed line shows the approximate level of the medians in the control samples. Boxplots were produced in R using the ggplot2 package.

As expected, the antibiotic treatment provided the same effect as in Figure 4d (Figure 6a). After 7 days of antibiotic administration, six volunteers were allowed to recover, and metagenomes of their gut were sampled after 56 days. Only bacteria of the third group approached their control level in the human intestine (Figure 6b). Twenty-eight days of probiotic therapy applied to five individuals caused an apparently more pronounced effect compared to the antibiotic-treated samples (Figure 6c). However, the variability of the *L. paracasei* response to antibiotic/probiotic administration made the observed changes statistically insignificant. Thus, at the next step we visualized these changes individually for all strains in all metagenomes.

### 3.7. Tracking Individual Changes in the Presence of L. paracasei Strains in Response to Antibiotic Administration

**Figure 7:**
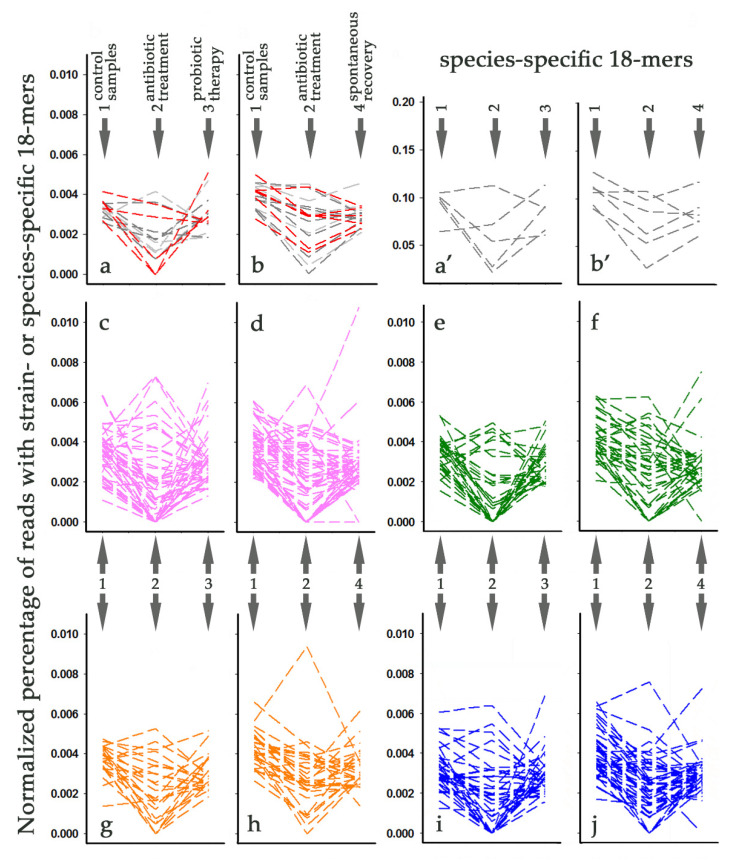
Visualization of individual changes in the abundance of *L. paracasei* strains in response to antibiotic administration (time point 2) and subsequent recovery after 56 days with (time point 3 in panels (**a**,**a’**,**c**,**e**,**g**,**i**)) or without (time point 4 in panels (**b**,**b’**,**d**,**f**,**h**,**j**)) probiotics. Line plots in panels (**a’**,**b’**) show data obtained with species-specific barcodes. All other panels demonstrate the time course of changes in 11 intestinal microbiomes obtained with strain-specific sets of 18-mers. The order of the samples and their specifications are shown in panels (**a**,**b**). The color code is the same as in the previous figures.

Figure 7a,b shows the pattern of changes in the multiplicity of bacteria containing marker 18-mers of *L. paracasei* VKM B-1144, NSMJ15, and NFF04 strains in 11 metagenomes. The same as in Figure 6, plots are combined into two groups, corresponding to samples obtained from individuals later either subjected to probiotic therapy (Figure 7a) or not (Figure 7b). In most cases, we observed an expected decrease in the number of strain-specific 18-mers or an absence of significant changes after antibiotic intake. However, barcodes of the *L. paracasei* NSMJ15 genome demonstrated an unusual increase in multiplicity. Many other examples of antibiotic-mediated stimulation were found for bacteria of all phylogroups (Figure 7b–j). This apparently disagrees with the volcano plot in Figure 5a, indicating a statistically significant decrease in the number of species-specific barcodes of all genomes in antibiotic-treated samples. Therefore, we checked the possibility that this difference is due to the specificity of strain-specific barcodes (Figure 6 and Figure 7). However, visualizing individual changes with species-specific sets of marker 18-mers, we observed the same alterations as in the case with strain-specific barcodes (exemplified in Figure 7a’,b’ for *L. paracasei* NSMJ15). Thus, it became clear that individual diversity is masked in volcano plots when averaged over 11 metagenomes.

All strains but one (*L. paracasei* CAUH35, Group IV) showed antibiotic-mediated augmentation in 1–4 of 11 microbiomes, and there is only one metagenome, where no such effect was detected (ERR2750509). Moreover, in six of the remaining 10 metagenomes, exemplifying the consequence of antibiotic administration and containing bacteria positively responding to this treatment, we were unable to detect the presence of lactobacilli with barcodes corresponding to 1–18 other strains. Thus, it is likely that the adaptation of microbiomes to drugs can be carried out at the level of intraspecific diversification, which was not expected *a priori*.

### 3.8. Tracking Individual Changes in the Presence of L. paracasei Strains in Response to Probiotic Therapy and Spontaneous Recovery

The average abundance of *L. paracasei* VKM B-1144 relatives increased after probiotic therapy (Figure 6c), and in one metagenome their presence (0.0051%) was even higher than before the administration of ciprofloxacin and metronidazole (0.0027%), although it decreased to zero after their intake (Figure 7a). A response with suppression and almost full recovery of these bacteria was observed in two other microbiomes (Figure 7a). However, the percentage of *L. paracasei* VKM B-1144 relatives in the remaining two samples reduced as expected in response to antibiotics, but decreased even more after probiotic therapy (Figure 7a).

The same behavior in one or two metagenomes was demonstrated by relatives of 16 other strains belonging to different phylogroups (Figure 7c,e,g,i). Among spontaneously recovered microbiomes, the number of strains with this type of change turned out to be higher (26), and in four cases (phylogroups I, II, IV), the final reduction reached zero (Figure 7d,f,h,j), which was not observed for the samples collected after probiotic therapy (Figure 7a,c,e,g,i). Almost all strains with a positive response to antibiotics tended to recover to the control level both after probiotic administration and without it. However, strains NJ (Figure 7c), TMW 1.1434 (Figure 7g), JCM 8130 (Figure 7i), ATCC 334 and FAM18149 (Figure 7j) each showed a further increase in a single metagenome. This may indicate antibiotic-mediated rearrangement in the host microbiome. In any case, visualization of individual changes in the percentage of reads containing strain-specific 18-mer codes allowed us to reveal four different scenarios used by microbiomes to withstand antibiotics.

## 4. Discussion

Among lactic bacteria, *L. paracasei* and its close relatives are considered as promising components of probiotics. Studies in animals and humans conducted at cell and organismic levels have shown dozens of its strains to possess beneficial properties for the host [53,54,55,56]. For instance, *L. paracasei* strain L1 turned out to be able to improve the gut microbiota in chickens and promoted their growth [53]; *L. paracasei* NCC2461 can attenuate antibiotic induced visceral hypersensitivity in mice [54]. Suzuki and co-authors [55] found *L. paracasei* KW3110 to inhibit NLRP3 inflammasome activation in macrophages from BALB/c mice, and this effect was shown to be IL-10-dependent. The same strain administered *per os* reduced monosodium urate crystal-induced peritoneal inflammation in C57BL/6 mice in vivo and its continuous intake mitigated insulin resistance caused by a high fat diet. In another work, Chondrou et al. [56] demonstrated immunomodulatory potential of *L. paracasei* K5, which promoted upregulation of interleukins IL-1α, ΙL-1β, IL-6, tumor necrosis factor-alpha, as well as some other products related to carcinogenesis, in the human cell line Caco-2. There are several other examples in the review by Vuong and Hsiao [50]. Thus, it is reasonable that bacteria of this species are used in probiotic compositions, although not so often as *Limosilactobacillus reuteri* or *Lacticasebacillus rhamnosus.* The newly characterized strain of *L. paracasei* VKM B-1144 may have an advantage as a probiotic due to its potentially high ability to colonize the intestinal epithelium.

Relatively rare use of *L. paracasei* in probiotic cocktails may partly be due to the not entirely clear phylogeny of *Lacticaseibacillus* genus bacteria, and one of the main results of this study is the first classification of the *L. paracasei* species at the intraspecific level. Revealing three stable phylogroups I, II and IV based on the set of complete genomes, we also detected subgroups in the phylogroup III and an additional clade between groups I and II, when the set, extended by yet unassembled genomes, was analyzed. This is important, because based on hundreds of datasets characterizing intestinal microbiomes, it is usually accepted that their complex species/genus composition can quickly adapt in response to dietary shifts or changes in the physiological state of the host. On the other hand, microbiomes can maintain enterotypes that were optimized in individuals during their development. Thus, it is generally believed that the combination of plasticity and stability is realized at the level of species diversity, while pathogenesis is caused by expansion of a certain strain, as was the case with the *E. coli* serotype O104:H4 [57].

Since the pathogenicity of individual strains largely depends on their evolutionary origin, information on intraspecific diversity is becoming more and more in demand, which requires the development and introduction of special tools into the routine practice of bioinformatics analysis. Here we used a *k*-mer-based approach, which allows the revelation of bacterial strains in natural biota and performing phylotyping without multiple sequence alignment of conserved orthologous proteins or genes. We demonstrated its heuristic efficiency by identifying three species of *L. paracasei* previously/recently attributed to *L. casei*, which are difficult to distinguish based on classical phylotyping by 16S rRNA due to the close relationship of these species.

For taxonomic analysis, we selected four datasets with different probabilities of having changes in the abundance level of *L. paracasei.* In our previous paper [5] we revealed an increase in the presence of *E. coli* in the intestinal microbiota of Crohn’s disease patients, and this effect turned out to be phylogroup dependent. Here we did not observe statistically significant changes in samples taken from Crohn’s disease patients, although some suppression of the presence of *L. paracasei* was detected (Figure 4). The main difference from the data obtained for *E. coli* lies in an approximately equal distribution of different *L. paracasei* strains (their relatives) in all the metagenomes. Since we dealt with *k*-mers absent in all complete bacterial genomes except the genomes of *L. paracasei*, the individual barcodes overlapped, and similar 18-mers contributed to the calculated percentage, probably masking the difference, but deviation from the control samples was negligible, so we did not continue analysis of this data set.

Two other sets of metadata, reliably show an absence of difference in *L. paracasei* abundance in the microbiota of healthy individuals and patients with ASD, as well as in response to an 8-week isocaloric dietary intervention with a Mediterranean diet used by obese persons. The fourth set of 33 metagenomes [34], on the contrary, allowed the revelation of a statistically significant and apparently phylogroup-dependent reduction of *L. paracasei* abundance in the gut microflora in response to antibiotic treatment (Figure 5a).

Probably, the most important and hardly expected observation was a very slow recovery of microbiota after antibiotic treatment (Figure 6b,c). This contradicts the expected plasticity of microbiomes, if it is not only due to the specific selection of antibiotic-naïve volunteers as models. Decreased levels of lactobacilli in their intestine may be a stage of a normal adaptive response, which might be preserved in the future.

In order to characterize the recovery in more detail, for the first time we implemented strain-specific barcodes, but did not observe significant differences between the phylogroups (Figure 6c), all of which showed unreliable changes in response to probiotic therapy. Moreover, only the relatives of *L. paracasei* strain TD062 from Group IV and ZY-1 from Group I statistically significantly accumulated after probiotic intake (*p* = 0.008 and 0.032, respectively). The next two effectively recovered bacteria were related to *L. paracasei* strain JCM 8130 from Group IV and the singleton NSMJ15 (*p* = 0.056 and 0.095, respectively). Since all these bacteria belong to different phylogroups, it is unlikely that their more stable recovery can be explained by the mere presence of *L. paracasei* BAA-52 bacteria in the probiotic cocktail [58] (its phylogroup is unknown, because the genome is absent in the NCBI database). However, within the framework of this study, we received a reasonable explanation for the absence of statistically significant changes in the responses of different phylogroups to antibiotics or probiotics. It is based on different scenarios used by bacteria in different metagenomes to adapt to these agents. Thus, in response to antibiotics, *L. paracasei* bacteria can both increase and decrease their content in metagenomes and usually tend to restore the initial level upon recovery. Hence, in many cases, the changes caused by antibiotics even appeared to strengthen after 56 days of recovery. To our knowledge, this is the first indication of such complex dynamics of antibiotic-induced changes. The fact that relatives of the same strain often behaved differently in different metagenomes, while in one metagenome (ERR2750041) we observed an antibiotic-mediated increase for relatives of many strains, indicates that the implementation of different growth scenarios by bacteria depends on the enterotype/composition of the intestinal microflora, which must be taken into account when carrying out probiotic therapy.

## Figures and Tables

**Table 1:** List of *Lacticaseibacillus* genomes used for intrageneric phylogenetic analysis.

N	Name of Strain	Phylo- Group	Number of Unique 18-Mers Absent in the Genomes of Other Genera and Plasmids	N	Name of Strain	Phylo- Group	Number of Unique 18-Mers Absent in the Genomes of Other Genera and Plasmids
***L. paracasei***				40	EG9	IV	1,243,018
1	MGB0747	I	1,342,841	41	N1115	IV	1,235,840
2	MGB0761	I	1,342,893	42	KL1	IV	1,234,653
3	L9	I	1,326,474	43	JCM 8130	IV	1,286,351
4	MGYG-HGUT-02388	I	1,326,474	44	MGB0734	-	1,327,753
5	NJ	I	1,335,035	45	NFFJ04	-	1,287,217
6	347–16	I	1,354,652	46	VKM B-1144	-	1,274,372
7	CACC 566	I	1,354,718	47	NSMJ15	-	1,208,720
8	Lp02	I	1,307,893	***L. casei***			
9	IJH-SONE68	I	1,316,002	48	ATCC 393		1,216,652
10	IIA	I	1,334,702	49	LC5		1,398,284
11	10266	I	1,306,215	***L. zeae***			
12	ZY-1	I	1,354,745	50	CECT 9104 (formerly *L. casei*)		1,326,946
13	LC355	I	1,311,398	***L. manihotivorans***			
14	SRCM103299	I	1,333,804	51	LM010		1,381,360
15	HDS-01	I	1,310,186	***L. rhamnosus***			
16	HD1–7	I	1,310,389	52	4B15		1,357,067
17	Zhang	II	1,267,650	53	GG		1,357,107
18	LOCK919	II	1,373,093	54	BIO6870		1,356,905
19	ZFM54	II	1,310,426	55	LR-B1		1,357,098
20	TK1501	II	1,289,097	56	WQ2		1,351,500
21	12A (annotated as *L. casei*)	**II**	1,301,297	57	LRB		1,333,164
22	8700:2	II	1,319,012	58	hsryfm 1301		1,352,189
23	Lpc10	II	1,345,201	59	DSM 14870		1,346,181
24	MGB0245	III	1,331,017	60	IDCC 3201		1,323,375
25	MGB0625	III	1,391,072	61	BFE5264		1,338,512
26	TK-P4A	III	1,352,170	62	1–0320		1,350,218
27	TMW 1–1434	III	1,323,010	63	LR5		1,370,793
28	IBB3423	III	1,400,224	64	LOCK900		1,332,726
29	CBA3611	III	1,316,237	65	Pen		1,329,920
30	LC2W	III	1,316,047	66	BPL5		1,392,367
31	AO356 (7112–2)	III	1,310,307	67	ATCC 8530		1,362,386
32	BL23 (formerly *L. casei*)	III	1,337,551	68	Lc 705		1,377,678
33	W56 (formerly *L. casei*)	III	1,337,353	69	ASCC 290		1,357,004
34	BD-II	III	1,337,137	70	SCT-10–10-60		1,377,318
35	TCS	III	1,337,717	71	ATCC 11443		1,377,473
36	TD 062	IV	1,129,425	72	LOCK908		1,377,399
37	FAM18149	IV	1,162,779	73	NCTC13764		1,377,359
38	CAUH35	IV	1,187,210	74	BIO5326		1,377,176
39	ATCC 334	IV	1,229,640	75	NCTC13710		1,377,380

**Table 2:** List of *L. paracasei* strains traced in human metagenomes.

N	Strain ^1^	PG ^2^	Number of		N	Strain		Number of	
			Species- Specific 18-Mers	Strain-Specific 18-Mers			**PG**	Species- Specific 18-Mers	Strain-Specific 18-Mers
1	MGB0747	**I**	1,201,260	83	25	MGB0245	**III**	1,184,705	**50,602**
2	MGB0761	**I**	1,201,306	81	26	MGB0625	**III**	1,228,586	**48,092**
3	L9	**I**	1,186,502	0	27	TK-P4A	**III**	1,212,483	**86,180**
4	MGYG-HGUT	**I**	1,186,506	0	28	TMW 1.1434	**III**	1,199,252	**25,090**
5	NJ	**I**	1,188,566	**17,470**	29	IBB3423	**III**	1,250,212	**38,367**
6	347–16	**I**	1,207,017	130	30	CBA3611	**III**	1,188,329	89
7	CACC 566	**I**	1,207,087	216	31	LC2W	**III**	1,188,152	38
8	Lp02	**I**	1,177,634	**11,840**	32	subsp. 7112–2	**III**	1,182,816	121
9	IJH-SONE68	**I**	1,183,896	**26,679**	33	BL23	**III**	1,199,366	427
10	IIA	**I**	1,198,699	**16,607**	34	W56	**III**	1,199,237	571
11	subsp 10266	**I**	1,172,488	**25,784**	35	BD-II	**III**	1,199,063	144
12	ZY-1	**I**	1,216,121	**46,665**	36	TCS	**III**	1,199,506	244
13	LC355	**I**	1,179,984	**17,903**	37	NFFJ04	-	1,155,507	**71,319**
14	SRCM 103299	**I**	1,194,448	**23,021**	38	NSMJ15	-	1,060,651	**77,519**
15	HD1.7	**I**	1,201,260	83	39	VKM B-1144	-	1,100,024	**73,388**
16	HDS-01	**I**	1,201,306	81	40	TD 062	**IV**	1,000,877	**22,825**
17	MGB0734	-	1,178,026	**65,524**	41	FAM18149	**IV**	1,028,072	**28,405**
18	Zhang	**II**	1,143,566	8885	42	CAUH35	**IV**	1,052,575	**38,375**
19	LOCK919	**II**	1,230,630	**28,394**	43	ATCC 334	**IV**	1,143,681	**38,932**
20	ZFM54	**II**	1,169,160	**22,145**	44	EG9	**IV**	1,093,799	**37,831**
21	TK1501	**II**	1,154,098	**19,871**	45	N1115	**IV**	1,105,817	**25,748**
22	12A	**II**	1,103,714	**28,753**	46	KL1	**IV**	1,165,966	**20,265**
23	subsp. 8700:2	**II**	1,184,388	**23,564**	47	JCM 8130	**IV**	1,134,441	**43,983**
24	Lpc10	**II**	1,179,984	**39,486**					

^1^ Strains indicated in bold were used for species-specific and strain-specific analysis. ^2^ Phylogroup.

## Data Availability

Assembled and annotated genome of *L. paracasei* VKM B-1144 is available in NCBI (BioProject PRJNA779281).

## Supplementary Materials

The following are available online at https://www.mdpi.com/article/10.3390/life11111246/s1: Figure S1. Phylogenetic tree inferred from a pairwise distance matrix for 222 sets of 18-mers unique to the species *L. paracasei*; Table S1. Metagenomes used for taxonomic analysis.; Table S2. List including 74 complete genomes of *Lacticaseibacillus* from the NCBI GenBank; Table S3. List of 175 additional *L. paracasei* genomes from the NCBI GenBank (assembled in contigs and scaffolds) used for intraspecific phylogenetic analysis.
