# Supplementary material for: Lacticaseibacillus paracasei: Occurrence in the Human Gut Microbiota and K-Mer-Based Assessment of Intraspecies Diversity
Source: Life (Basel). 2021 Nov 17;11(11):1246. doi: 10.3390/life11111246 (PMC8620312; doi:10.3390/life11111246)
Supplement: Supplementary file 1 [file Supplementary Figure 1.pdf › Supplementary Figure 1.pdf]

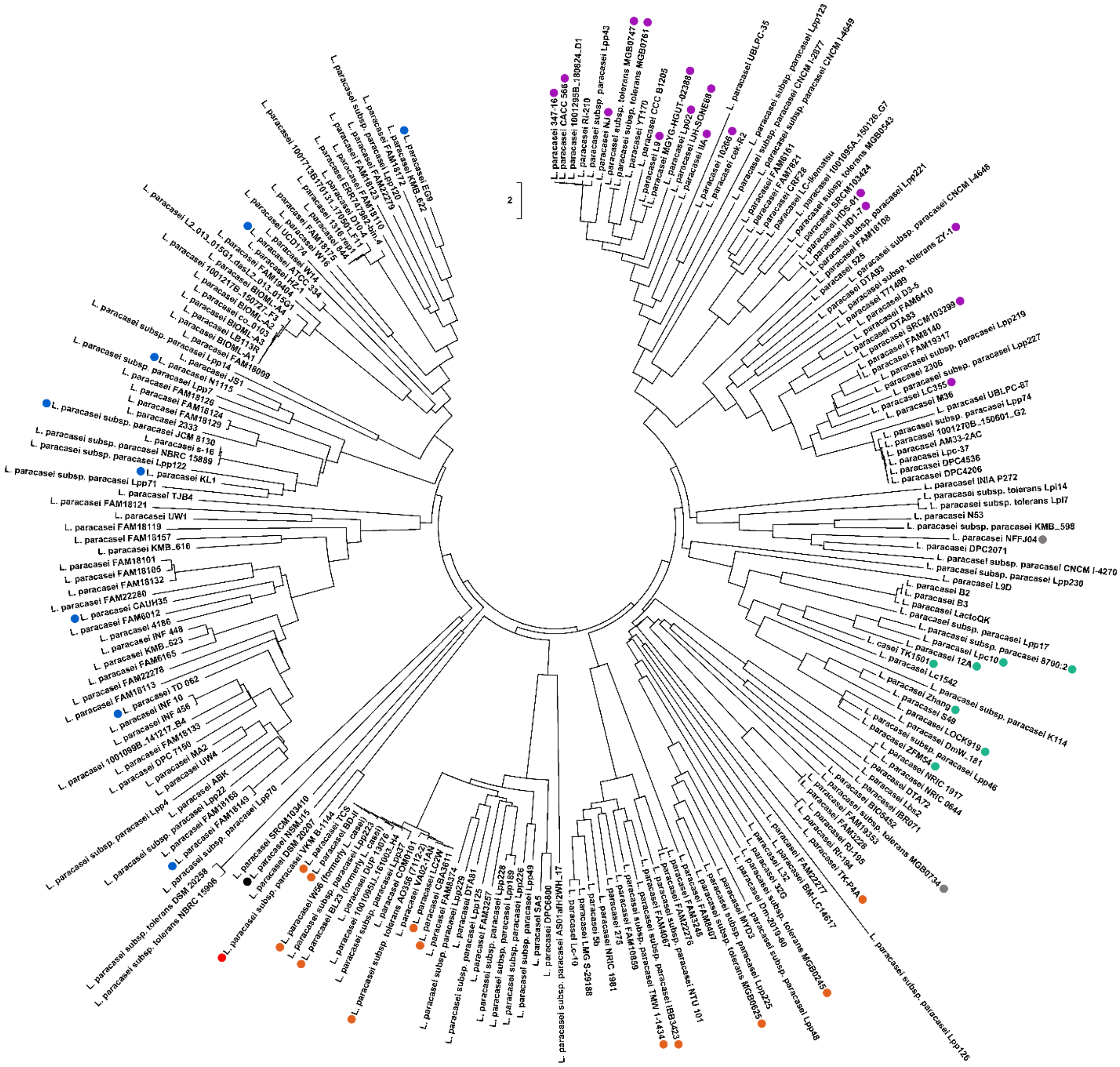

Supplementary Figure S1. Phylogenetic tree inferred from a pairwise distance matrix for 222 sets of 18-mers unique to the species *L. paracasei.* The tree was constructed in MEGA X software using the neighbor-joining method. The allocation of 47 genomes to the phylogroups identified in Figures 2 and 3, is indicated by colored circles. Scale bar shows the Sørensen distance as a percentage.
