# Supplementary material for: Lacticaseibacillus paracasei: Occurrence in the Human Gut Microbiota and K-Mer-Based Assessment of Intraspecies Diversity
Source: Life (Basel). 2021 Nov 17;11(11):1246. doi: 10.3390/life11111246 (PMC8620312; doi:10.3390/life11111246)
Supplement: Supplementary file 1 [file life-11-01246-s001.zip › Supplementary Table 1.pdf]

Supplementary Table S1. Metagenomes used for taxonomic analysis.

Project/Accession number	Number of reads passed QC	Reference	Note
**Metagenomes of healthy volunteers and patients with autism**			
PRJEB23052/ERR2608620	6509332	1, [31][1]	Healthy
PRJEB23052/ERR2608621	6315097	1, [31]	Healthy
PRJEB23052/ERR2608622	7392754	1, [31]	Healthy
PRJEB23052/ERR2608623	5185236	1, [31]	Healthy
PRJEB23052/ERR2608624	5163476	1, [31]	Healthy
PRJEB23052/ERR2608625	5276922	1, [31]	Healthy
PRJEB23052/ERR2608626	5115260	1, [31]	Healthy
PRJEB23052/ERR2608627	5091624	1, [31]	Healthy
PRJEB23052/ERR2608628	5232997	1, [31]	Healthy
PRJEB23052/ERR2608629	6306590	1, [31]	Healthy
PRJEB23052/ERR2608630	2636570	1, [31]	Autism
PRJEB23052/ERR2608631	3489522	1, [31]	Autism
PRJEB23052/ERR2608632	2926848	1, [31]	Autism
PRJEB23052/ERR2608633	5531988	1, [31]	Autism
PRJEB23052/ERR2608634	1614156	1, [31]	Autism
PRJEB23052/ERR2608635	1250119	1, [31]	Autism
PRJEB23052/ERR2608636	3774928	1, [31]	Autism
PRJEB23052/ERR2608637	3214364	1, [31]	Autism
PRJEB23052/ERR2608638	4403534	1, [31]	Autism
PRJEB23052/ERR2608639	5728095	1, [31]	Autism
**Metagenomes of healthy volunteers and patients with Chron's disease**			
PRJNA290380/SRR3582134	9565987	2, [32]	Healthy
PRJNA290380/SRR3582142	8037728	2, [32]	Healthy
PRJNA290380/SRR3582158	12045393	2, [32]	Healthy
PRJNA290380/SRR3582179	27779132	2, [32]	Healthy
PRJNA290380/SRR3582131	10553860	2, [32]	Crohn's disease
PRJNA290380/SRR3582133	4045307	2, [32]	Crohn's disease
PRJNA290380/SRR3582135	7422718	2, [32]	Crohn's disease
PRJNA290380/SRR3582143	7981819	2, [32]	Crohn's disease
PRJNA290380/SRR3582147	3056504	2, [32]	Crohn's disease
PRJNA290380/SRR3582151	10715446	2, [32]	Crohn's disease
PRJNA290380/SRR3582153	15284783	2, [32]	Crohn's disease
PRJNA290380/SRR3582154	5964329	2, [32]	Crohn's disease
PRJNA290380/SRR3582159	19754882	2, [32]	Crohn's disease
PRJNA290380/SRR3582163	13839249	2, [32]	Crohn's disease
PRJNA290380/SRR3582166	14180580	2, [32]	Crohn's disease
PRJNA290380/SRR3582168	13243423	2, [32]	Crohn's disease
PRJNA290380/SRR3582173	6585074	2, [32]	Crohn's disease
PRJNA290380/SRR3582175	5246794	2, [32]	Crohn's disease
PRJNA290380/SRR3582180	9365493	2, [32]	Crohn's disease
**Metagenomes of obese volunteers before and after adherence to dietary restrictions[2]**			
PRJEB33500/ERR3857002	1959995	3, [33]	Obesity
PRJEB33500/ERR3857012	1826406		8 weeks diet

PRJEB33500/ERR3857254	1874205	3, [33]	Obesity
PRJEB33500/ERR3857017	2229601		8 weeks diet
PRJEB33500/ERR3857037	1916084	3, [33]	Obesity
PRJEB33500/ERR3857027	1497878		8 weeks diet
PRJEB33500/ERR3857100	2925880	3, [33]	Obesity
PRJEB33500/ERR3857095	1871536		8 weeks diet
PRJEB33500/ERR3857146	2585136	3, [33]	Obesity
PRJEB33500/ERR3857109	1759546		8 weeks diet
PRJEB33500/ERR3857205	2543813	3, [33]	Obesity
PRJEB33500/ERR3857155	2212505		8 weeks diet
PRJEB33500/ERR3857215	2303995	3, [33]	Obesity
PRJEB33500/ERR3857175	1528328		8 weeks diet
PRJEB33500/ERR3857170	1923341	3, [33]	Obesity
PRJEB33500/ERR3857190	2278692		8 weeks diet
PRJEB33500/ERR3857279	2311426	3, [33]	Obesity
PRJEB33500/ERR3857263	1540527		8 weeks diet
PRJEB33500/ERR3857305	1909621	3, [33]	Obesity
PRJEB33500/ERR3857290	1594468		8 weeks diet
PRJEB33500/ERR3857350	1527538	3, [33]	Obesity
PRJEB33500/ERR3857370	2698429		8 weeks diet
PRJEB33500/ERR3857380	1419732	3, [33]	Obesity
PRJEB33500/ERR3857365	2182872		8 weeks diet
PRJEB33500/ERR3857423	1453389	3, [33]	Obesity
PRJEB33500/ERR3857413	1812433		8 weeks diet
PRJEB33500/ERR3857532	1490011	3, [33]	Obesity
PRJEB33500/ERR3857540	2367644		8 weeks diet
PRJEB33500/ERR3857586	3709986	3, [33]	Obesity
PRJEB33500/ERR3857598	2029092		8 weeks diet
PRJEB33500/ERR3857642	2099219	3, [33]	Obesity
PRJEB33500/ERR3857674	2292734		8 weeks diet
PRJEB33500/ERR3857795	460416	3, [33]	Obesity
PRJEB33500/ERR3857803	333362		8 weeks diet
PRJEB33500/ERR3857816	646093	3, [33]	Obesity
PRJEB33500/ERR3857807	518009		8 weeks diet
PRJEB33500/ERR3857895	2699677	3, [33]	Obesity
PRJEB33500/ERR3857969	4012580		8 weeks diet
PRJEB33500/ERR3857901	4518911	3, [33]	Obesity
PRJEB33500/ERR3857947	5372218		8 weeks diet
PRJEB33500/ERR3857907	3689335	3, [33]	Obesity
PRJEB33500/ERR3857905	4840823		8 weeks diet
PRJEB33500/ERR3857945	3084073	3, [33]	Obesity
PRJEB33500/ERR3857954	3249622		8 weeks diet
PRJEB33500/ERR3857949	3278467	3, [33]	Obesity
PRJEB33500/ERR3857960	3643305		8 weeks diet
PRJEB33500/ERR3857977	3087302	3, [33]	Obesity
PRJEB33500/ERR3857975	3644496		8 weeks diet
PRJEB33500/ERR3857987	4184598	3, [33]	Obesity
PRJEB33500/ERR3857989	4184598		8 weeks diet

Metagenomes of antibiotic-naïve individuals subjected to sequential antibiotic and probiotic treatment[2]

PRJEB28097/ERR2749975	538322		Healthy
PRJEB28097/ERR2749984	87260	4, [34]	Antibiotic
PRJEB28097/ERR2750001	425427		Probiotic
PRJEB28097/ERR2750032	989767		Healthy
PRJEB28097/ERR2750041	284990	4, [34]	Antibiotic
PRJEB28097/ERR2750065	2058912		Probiotic
PRJEB28097/ERR2750067	1119488		Healthy
PRJEB28097/ERR2750080	60023	4, [34]	Antibiotic
PRJEB28097/ERR2750102	1112280		Probiotic
PRJEB28097/ERR2750170	1126700		Healthy
PRJEB28097/ERR2750183	200757	4, [34]	Antibiotic
PRJEB28097/ERR2750192	1664514		Probiotic
PRJEB28097/ERR2750195	1529932		Healthy
PRJEB28097/ERR2750201	558447	4, [34]	Antibiotic
PRJEB28097/ERR2750216	2210574		Probiotic
PRJEB28097/ERR2750326	917099		Healthy
PRJEB28097/ERR2750336	617510	4, [34]	Antibiotic
PRJEB28097/ERR2750351	2787835		SR[3]
PRJEB28097/ERR2750353	1070291		Healthy
PRJEB28097/ERR2750365	188404	4, [34]	Antibiotic
PRJEB28097/ERR2750387	2724814		SR
PRJEB28097/ERR2750390	1129759		Healthy
PRJEB28097/ERR2750403	499227	4, [34]	Antibiotic
PRJEB28097/ERR2750426	2397303		SR
PRJEB28097/ERR2750429	738611		Healthy
PRJEB28097/ERR2750440	301194	4, [34]	Antibiotic
PRJEB28097/ERR2750461	104661		SR
PRJEB28097/ERR2750499	1083440		Healthy
PRJEB28097/ERR2750509	120275	4, [34]	Antibiotic
PRJEB28097/ERR2750527	2356092		SR
PRJEB28097/ERR2750530	954529		Healthy
PRJEB28097/ERR2750542	277826	4, [34]	Antibiotic
PRJEB28097/ERR2750566	1434943		SR

[1] Ciphers in the brackets indicate reference number in the main text

[2] Directly compared metagenomes grouped together.

[3] Spontaneously recovered (SR)

References used in Supplementary Table S1

1. Wang M. et al. Alterations in Gut Glutamate Metabolism Associated with Changes in Gut Microbiota Composition in Children with Autism Spectrum Disorder // mSystems. mSystems, 2019. Vol. 4, № 1.

2. Vaughn B. et al. Increased Intestinal Microbial Diversity Following Fecal Microbiota Transplant for Active Crohn’s Disease // Inflamm. Bowel Dis. Inflamm Bowel Dis, 2016. Vol. 22, № 9. P. 2182–2190.

3. Meslier V. et al. Mediterranean diet intervention in overweight and obese subjects lowers plasma cholesterol and causes changes in the gut microbiome and metabolome independently of energy intake // Gut. Gut, 2020. Vol. 69, № 7. P. 1258–1268.

4. Montassier E. et al. Probiotics impact the antibiotic resistance gene reservoir along the human GI tract in a person-specific and antibiotic-dependent manner // Nat. Microbiol. Nat Microbiol, 2021. Vol. 6, № 8. P. 1043–1054.
