# Supplementary material for: Lacticaseibacillus paracasei: Occurrence in the Human Gut Microbiota and K-Mer-Based Assessment of Intraspecies Diversity
Source: Life (Basel). 2021 Nov 17;11(11):1246. doi: 10.3390/life11111246 (PMC8620312; doi:10.3390/life11111246)
Supplement: Supplementary file 1 [file life-11-01246-s001.zip › Supplementary Table 2.pdf]

Table S2. List including 74 complete genomes of *Lacticaseibacillus* from NCBI GenBank.

Acsession number	Strain	Genome length, bp	GC-content, %
CP031785.1	*L. paracasei* 10266	3012260	46.4
CP006690.1	*L. casei* 12A	2907892	46.4
CP052065.1	*L. paracasei* 347-16	3102350	46.3
CP000423.1	*L. paracasei* ATCC 334	2895264	46.6
CP002618.1	*L. paracasei* BD-II	3069926	46.3
FM177140.1	*L. paracasei* BL23 (formerly *L. casei*)	3079196	46.3
CP048003.1	*L. paracasei* CACC 566	3123521	46.3
CP012187.1	*L. paracasei* CAUH35	2770411	46.6
CP041657.1	*L. paracasei* CBA3611	3035735	46.4
CP029546.1	*L. paracasei* EG9	2927257	46.6
CP017261.1	*L. paracasei* FAM18149	2710941	46.7
CP025582.1	*L. paracasei* HD1-7	3039280	46.4
CP026097.1	*L. paracasei* HDS-01	3038767	46.4
CP014985.1	*L. paracasei* IIA	3055892	46.3
AP018392.1	*L. paracasei* IJH-SONE68	3084917	46.4
CP013921.1	*L. paracasei* KL1	2918888	46.6
CP012148.1	*L. paracasei* L9	3076437	46.3
CP002616.1	*L. paracasei* LC2W	3039042	46.4
CP029536.1	*L. paracasei* LC355	3044973	46.5
CP005486.1	*L. paracasei* LOCK919	3113601	46.2
CP039707.1	*L. paracasei* Lp02	2991176	46.3
CP029686.1	*L. paracasei* Lpc10	3052122	46.3
LR698988.1	*L. paracasei* MGYG-HGUT-02388	3076437	46.3
CP007122.1	*L. paracasei* N1115	2938059	46.6
CP050500.1	*L. paracasei* NFFJ04	2983945	46.5
CP041944.1	*L. paracasei* NJ	3083412	46.4
CP049324.1	*L. paracasei* NSMJ15	2791277	46.6
CP035563.1	*L. paracasei* SRCM103299	3081420	46.4
CP045567.1	*L. paracasei* TK-P4A	3020185	46.4
CP001084.2	*L. paracasei* Zhang	2861969	46.5
CP002391.1	*L. paracasei* subsp. *paracasei* 8700:2	2939026	46.4
CP022954.1	*L. paracasei* subsp. *paracasei* IBB3423	3183386	46.3
AP012541.1	*L. paracasei* subsp. *paracasei* JCM 8130	2995875	46.6
CP016355.1	*L. paracasei* subsp. *paracasei* TMW 1-1434	3063698	46.4
CP025499.1	*L. paracasei* subsp. *tolerans* AO356 (7112-2)	3022095	46.4
CP064299.1	*L. paracasei* subsp. *tolerans* MGB0245	3042298	46.4
CP064311.1	*L. paracasei* subsp. *tolerans* MGB0625	3202250	46.3
CP064304.1	*L. paracasei* subsp. *tolerans* MGB0734	3079306	46.4
CP064314.1	*L. paracasei* subsp. *tolerans* MGB0747	3071809	46.3
CP064316.1	*L. paracasei* subsp. *tolerans* MGB0761	3086705	46.3
CP065154.1	*L. paracasei* subsp. *tolerans* ZY-1	3148894	46.4
CP038153.1	*L. paracasei* TCS	3077701	46.3
CP044361.1	*L. paracasei* TD 062	2667255	46.7
CP017716.1	*L. paracasei* TK1501	2942538	46.5
HE970764.1	*L. paracasei* W56 (formerly *L. casei*)	3075780	46.3
CP032637.1	*L. paracasei* ZFM54	3015887	46.4
CP017065.1	*L. casei* LC5	3132867	47.9
AP012544.1	*L. casei* subsp. *casei* ATCC 393	2924929	47.9
LS991421.1	*L. zeae* CECT 9104 (formerly *L. casei*)	3045762	48.0

CP045068.1	*L. manihotivorans* LM010	3395916	47.8
CP040780.1	*L. rhamnosus* 1-0320	2941522	46.7
CP021426.1	*L. rhamnosus* 4B15	3047840	46.6
CP014645.1	*L. rhamnosus* ASCC 290	2903890	46.7
CP022109.1	*L. rhamnosus* ATCC 11443	2990082	46.8
CP003094.1	*L. rhamnosus* ATCC 8530	2960339	46.8
CP014201.1	*L. rhamnosus* BFE5264	3068152	46.8
CP046267.1	*L. rhamnosus* BIO5326	2989570	46.8
CP044506.1	*L. rhamnosus* BIO6870	3006715	46.7
LT220504.1	*L. rhamnosus* BPL5	3024027	46.7
CP006804.1	*L. rhamnosus* DSM 14870	3013149	46.7
CP031290.1	*L. rhamnosus* GG	3010116	46.7
CP044228.1	*L. rhamnosus* hsryfm 1301	3022021	46.8
CP045531.1	*L. rhamnosus* IDCC 3201	3064363	46.7
FM179323.1	*L. rhamnosus* Lc 705	2968598	46.7
CP005484.1	*L. rhamnosus* LOCK900	2883376	46.8
CP005485.1	*L. rhamnosus* LOCK908	2990900	46.8
CP017063.1	*L. rhamnosus* LR5	2972590	46.7
CP016823.1	*L. rhamnosus* LRB	2934954	46.8
CP025428.1	*L. rhamnosus* LR-B1	3007503	46.7
LR134322.1	*L. rhamnosus* NCTC13710	2991048	46.8
LR134331.1	*L. rhamnosus* NCTC13764	2988387	46.8
CP020464.1	*L. rhamnosus* Pen	2884966	46.8
CP019305.1	*L. rhamnosus* SCT-10-10-60	2990071	46.8
CP020016.1	*L. rhamnosus* WQ2	2960737	45.9
