# Supplementary material for: Lacticaseibacillus paracasei: Occurrence in the Human Gut Microbiota and K-Mer-Based Assessment of Intraspecies Diversity
Source: Life (Basel). 2021 Nov 17;11(11):1246. doi: 10.3390/life11111246 (PMC8620312; doi:10.3390/life11111246)
Supplement: Supplementary file 1 [file life-11-01246-s001.zip › Supplementary Table 3.pdf]

Table S3. List of 175 additional *L. paracasei* genomes from NCBI GenBank (assemblied in contigs and scaffolds) used for intraspecific phylogenetic analysis.

	Acsession number	Strain
1	JADNKT010000000	*L. paracasei* 1001095A_150126_G7
2	JADMSR010000000	*L. paracasei* 1001095IJ_161003_H4
3	JADNFJ010000000	*L. paracasei* 1001099B_141217_B4
4	JADMOS010000000	*L. paracasei* 1001217B_150727_F3
5	JADOAX010000000	*L. paracasei* 1001270B_150601_G2
6	JADNCF010000000	*L. paracasei* 1001295B_180824_D1
7	JADOAW010000000	*L. paracasei* 1001713B170131_170501_F11
8	JVUB01000000	*L. paracasei* 1316.rep1
9	JAFEHM010000000	*L. paracasei* 2306
10	JAFEHK010000000	*L. paracasei* 2333
11	JVNI01000000	*L. paracasei* 275
12	AFYL01000000	*L. paracasei* 32G
13	JAFEHL010000000	*L. paracasei* 4186
14	JVDQ01000000	*L. paracasei* 525
15	AUYM01000000	*L. paracasei* 5b
16	JUQW01000000	*L. paracasei* 844
17	JAHLXM010000000	*L. paracasei* ABK
18	QVHW01000000	*L. paracasei* AM33-2AC
19	NXET01000000	*L. paracasei* B2
20	NXES01000000	*L. paracasei* B3
21	WBNY01000000	*L. paracasei* BIO5452
22	WKPH01000000	*L. paracasei* BIOML-A1
23	WKPG01000000	*L. paracasei* BIOML-A2
24	WKPF01000000	*L. paracasei* BIOML-A3
25	WKPE01000000	*L. paracasei* BIOML-A4
26	LTDP01000000	*L. paracasei* BM-LC14617
27	LGIY01000000	*L. paracasei* CCC B1205
28	JAIPUN010000000	*L. paracasei* cek-R2
29	RCYL01000000	*L. paracasei* co_0103
30	BAGT01000000	*L. paracasei* COM0101
31	AFYN01000000	*L. paracasei* CRF28
32	JAAFGQ010000000	*L. paracasei* D10-4
33	JAACXY010000000	*L. paracasei* D3-5
34	JAERKI010000000	*L. paracasei* Dm-2019-60
35	NDXH01000000	*L. paracasei* DmW_181
36	VLHQ01000000	*L. paracasei* DPC 7150
37	NCSN01000000	*L. paracasei* DPC2071
38	NCSO01000000	*L. paracasei* DPC4206
39	NCSP01000000	*L. paracasei* DPC4536
40	LNQD01000000	*L. paracasei* DPC6800
41	JYBD01000000	*L. paracasei* DSM 20207
42	WUJH01000000	*L. paracasei* DTA72
43	JAAVWK010000000	*L. paracasei* DTA81
44	QRBH01000000	*L. paracasei* DTA83
45	VTYT01000000	*L. paracasei* DTA93
46	PKQJ01000000	*L. paracasei* DUP 13076
47	CAJJVN010000000	*L. paracasei* ERR747982-bin.4
48	LKFC01000000	*L. paracasei* FAM10859
49	LKFD01000000	*L. paracasei* FAM18099

50	LKFE01000000	*L. paracasei* FAM18101
51	LKFF01000000	*L. paracasei* FAM18105
52	LKFG01000000	*L. paracasei* FAM18108
53	LKFH01000000	*L. paracasei* FAM18110
54	LKFI01000000	*L. paracasei* FAM18113
55	LKFJ01000000	*L. paracasei* FAM18119
56	LKFK01000000	*L. paracasei* FAM18121
57	LKFL01000000	*L. paracasei* FAM18123
58	LKFM01000000	*L. paracasei* FAM18124
59	LKFN01000000	*L. paracasei* FAM18126
60	LKFO01000000	*L. paracasei* FAM18129
61	LKFP01000000	*L. paracasei* FAM18132
62	LKFQ01000000	*L. paracasei* FAM18133
63	LKFS01000000	*L. paracasei* FAM18157
64	LKFT01000000	*L. paracasei* FAM18168
65	LKFU01000000	*L. paracasei* FAM18172
66	LKFV01000000	*L. paracasei* FAM18175
67	LKFW01000000	*L. paracasei* FAM19317
68	LKFX01000000	*L. paracasei* FAM19353
69	LKFY01000000	*L. paracasei* FAM19404
70	LKFZ01000000	*L. paracasei* FAM22276
71	LKGA01000000	*L. paracasei* FAM22277
72	LKGB01000000	*L. paracasei* FAM22278
73	LKGC01000000	*L. paracasei* FAM22279
74	LKGD01000000	*L. paracasei* FAM22280
75	LKGE01000000	*L. paracasei* FAM3228
76	LKGF01000000	*L. paracasei* FAM3248
77	LKGG01000000	*L. paracasei* FAM3257
78	LKGH01000000	*L. paracasei* FAM4067
79	LKGI01000000	*L. paracasei* FAM6012
80	LKGJ01000000	*L. paracasei* FAM6161
81	LKGK01000000	*L. paracasei* FAM6165
82	LKGL01000000	*L. paracasei* FAM6410
83	LKGM01000000	*L. paracasei* FAM7821
84	LKGN01000000	*L. paracasei* FAM8140
85	LKGO01000000	*L. paracasei* FAM8374
86	LKGP01000000	*L. paracasei* FAM8407
87	MOAG01000000	*L. paracasei* HZ-1
88	CAJHQN010000000	*L. paracasei* IBR071
89	JXRL01000000	*L. paracasei* INF 10
90	JXRJ01000000	*L. paracasei* INF 448
91	JXRK01000000	*L. paracasei* INF 456
92	CABFNE010000000	*L. paracasei* INIA P272
93	QHHM01000000	*L. paracasei* JS1
94	QMJP01000000	*L. paracasei* KMB_616
95	QMJV01000000	*L. paracasei* KMB_622
96	QMJW01000000	*L. paracasei* KMB_623
97	JAGZXG010000000	*L. paracasei* L2_013_015G1_dasL2_013_015G1
98	SEMI01000000	*L. paracasei* L32
99	MBTZ01000000	*L. paracasei* L9D
100	VTDA01000000	*L. paracasei* LactoQK
101	JAIFOL010000000	*L. paracasei* LB113R
102	JPKN03000000	*L. paracasei* Lbs2

103	AFYT01000000	*L. paracasei* Lc-10
104	LDEJ01000000	*L. paracasei* Lc1542
105	BDIT01000000	*L. paracasei* LC-Ikematsu
106	NIGW01000000	*L. paracasei* LMG S-29188
107	NOKL01000000	*L. paracasei* Lpc-37
108	AFYO01000000	*L. paracasei* M36
109	JAHLXL010000000	*L. paracasei* MA2
110	JAHHQA010000000	*L. paracasei* MYD3
111	JAHLOK010000000	*L. paracasei* N53
112	BAYM01000000	*L. paracasei* NRIC 0644
113	BAYN01000000	*L. paracasei* NRIC 1917
114	BAYO01000000	*L. paracasei* NRIC 1981
115	MKFZ01000000	*L. paracasei* RI-194
116	MKGA01000000	*L. paracasei* RI-195
117	MKFY01000000	*L. paracasei* RI-210
118	JACEIM010000000	*L. paracasei* s-16
119	SUMT01000000	*L. paracasei* S49
120	VCMK01000000	*L. paracasei* SA5
121	SCEM01000000	*L. paracasei* SRCM103410
122	SCEO01000000	*L. paracasei* SRCM103424
123	JAAXZG010000000	*L. paracasei* subsp. *paracasei* AS01afH2WH_17
124	ANLE01000000	*L. paracasei* subsp. *paracasei* CNCM I-2877
125	ANJX01000000	*L. paracasei* subsp. *paracasei* CNCM I-4270
126	ANKL01000000	*L. paracasei* subsp. *paracasei* CNCM I-4648
127	ANKH01000000	*L. paracasei* subsp. *paracasei* CNCM I-4649
128	WBOW01000000	*L. paracasei* subsp. *paracasei* K114
129	QMJF01000000	*L. paracasei* subsp. *paracasei* KMB_598
130	ANMK01000000	*L. paracasei* subsp. *paracasei* Lpp120
131	ANKW01000000	*L. paracasei* subsp. *paracasei* Lpp122
132	ANJW01000000	*L. paracasei* subsp. *paracasei* Lpp123
133	ANKM01000000	*L. paracasei* subsp. *paracasei* Lpp125
134	ANKC01000000	*L. paracasei* subsp. *paracasei* Lpp126
135	ANJZ01000000	*L. paracasei* subsp. *paracasei* Lpp14
136	ANMH01000000	*L. paracasei* subsp. *paracasei* Lpp17
137	ANJY01000000	*L. paracasei* subsp. *paracasei* Lpp189
138	ANJS01000000	*L. paracasei* subsp. *paracasei* Lpp219
139	ANMI01000000	*L. paracasei* subsp. *paracasei* Lpp22
140	ANKF01000000	*L. paracasei* subsp. *paracasei* Lpp221
141	ANML01000000	*L. paracasei* subsp. *paracasei* Lpp223
142	ANMM01000000	*L. paracasei* subsp. *paracasei* Lpp225
143	ANMN01000000	*L. paracasei* subsp. *paracasei* Lpp226
144	ANKK01000000	*L. paracasei* subsp. *paracasei* Lpp227
145	ANKA01000000	*L. paracasei* subsp. *paracasei* Lpp228
146	ANJT01000000	*L. paracasei* subsp. *paracasei* Lpp229
147	ANKY01000000	*L. paracasei* subsp. *paracasei* Lpp230
148	ANKG01000000	*L. paracasei* subsp. *paracasei* Lpp37
149	ANKE01000000	*L. paracasei* subsp. *paracasei* Lpp4
150	ANKI01000000	*L. paracasei* subsp. *paracasei* Lpp43
151	ANMJ01000000	*L. paracasei* subsp. *paracasei* Lpp46
152	ANLG01000000	*L. paracasei* subsp. *paracasei* Lpp48
153	ANKJ01000000	*L. paracasei* subsp. *paracasei* Lpp49
154	ANJV01000000	*L. paracasei* subsp. *paracasei* Lpp7
155	ANLF01000000	*L. paracasei* subsp. *paracasei* Lpp70

156	ANKD01000000	*L. paracasei* subsp. *paracasei* Lpp71
157	ANJU01000000	*L. paracasei* subsp. *paracasei* Lpp74
158	BJVL01000000	*L. paracasei* subsp. *paracasei* NBRC 15889
159	NPOB02000000	*L. paracasei* subsp. *paracasei* NTU 101
160	AYYJ01000000	*L. paracasei* subsp. *tolerans* DSM 20258
161	ANKB01000000	*L. paracasei* subsp. *tolerans* Lpl14
162	ANKV01000000	*L. paracasei* subsp. *tolerans* Lpl7
163	JADIXQ010000000	*L. paracasei* subsp. *tolerans* MGB0543
164	BJVO01000000	*L. paracasei* subsp. *tolerans* NBRC 15906
165	AFYP01000000	*L. paracasei* T71499
166	QXET01000000	*L. paracasei* TJB4
167	RCFI01000000	*L. paracasei* UBLPC-35
168	JADDXY010000000	*L. paracasei* UBLPC-87
169	AFYQ01000000	*L. paracasei* UCD174
170	AFYR01000000	*L. paracasei* UW1
171	AQVS01000000	*L. paracasei* UW4
172	JAAJBK010000000	*L. paracasei* VA02-1AN
173	MWVE01000000	*L. paracasei* W14
174	MWVD01000000	*L. paracasei* W16
175	JAFELK010000000	*L. paracasei* YT170
